# Increased Risk of Gout among Patients with Trisomy 21: Insights from a Population-Based Study of over 30,000 Patients with Trisomy 21

**DOI:** 10.31138/mjr.011225.alr

**Published:** 2026-06-16

**Authors:** Ahmed Abdelmaksoud, Ann Igoe, Mahmoud A. AbdAlnaeem, Eman A. Toraih, Bryan A. Roller, Kristin L. Kaelber, David C. Kaelber

**Affiliations:** 1 Department of Internal Medicine, School of Medicine, University of California, Riverside, CA, United States;; 2 School of Medicine, Case Western Reserve University, Cleveland, OH, United States;; 3 School of Medicine, Tulane University, New Orleans, LA, United States;; 4 Genetics Unit, Department of Histology and Cell Biology, Faculty of Medicine, Suez Canal University, Ismailia, Egypt;; 5 University of Minnesota, Minneapolis, MN, United States;; 6 Conner Integrative Health Network, University Hospitals of Cleveland, OH, United States;; 7 Centre for Clinical Informatics Research and Education, The MetroHealth System, Cleveland, OH, United States;; 8 Departments of Internal Medicine, Paediatrics, and Population and Quantitative Health Sciences, Case Western Reserve University, Cleveland, OH, United States

**Keywords:** rheumatoid arthritis, autoimmune diseases, gastrointestinal microbiome, immune system, inflammation, microbiota

## Abstract

**Background:**

Primary trisomy 21 is associated with an elevated incidence of conditions such as cardiac anomalies, hypothyroidism, type 1 diabetes mellitus, and auditory impairments. However, the potential association between trisomy 21 and gout remains insufficiently studied, with existing evidence largely limited to case reports. This study aimed to determine the prevalence of gout among individuals with trisomy 21 and compare it with those without trisomy 21 to evaluate a potential association and examine demographic factors influencing gout risk.

**Methods:**

This retrospective analysis utilised electronic health records from the US Collaborative Network within the TriNetX clinical research platform, representing over 100 million patients. We identified 30,171 individuals with trisomy 21 and compared them with 3,502,718 non-trisomy 21 controls using propensity score matching. Gout prevalence and relative risk (RR) with 95% confidence intervals (CI) were assessed between groups.

**Findings:**

Patients with trisomy 21 exhibited higher gout prevalence than controls (2.90% vs 1.05%, p < 0.0001). The relative risk of gout was 2.76 in individuals with trisomy 21 compared with controls (95% CI 2.43–3.14). Increased risk persisted across age, sex, and racial groups.

**Interpretation:**

These findings demonstrate an association between trisomy 21 and increased risk of gout. Further research is needed to understand the underlying mechanisms and inform targeted screening and management strategies in this population.

## KEY MESSAGES

- Hyperuricaemia has been shown to be commonly seen in patients with trisomy 21. However, the connection between clinical gout and trisomy 21 is only reported in a few case reports.
- Data from our study shows an association between trisomy 21 and the development of clinical gout in adult patients.
- Our findings support future research on mechanisms of this association and clinical preventive measures of gout-associated complications.

## INTRODUCTION

Gout is the most common inflammatory arthritis, affecting 3.9% of the US adult population, with an average age range of initial diagnosis between 40 and 69 years.^1^ The most recognised non-modifiable risk factors for gout development include increasing age, male sex, and African American race^1,3^ and the modifiable risk factors include diet, lifestyle, diuretic use, and the comorbidities associated with elevated cardiovascular risk, such as high body mass index and hypertension.^3–5^ Men outnumber women in all age groups, although the discrepancy decreases with increasing age.^3^ Risk factors associated with gouty flares in men are mostly related to the consumption of red meat, seafood, and alcohol, whereas gout flares in women are often secondary to diuretic use and/or concomitant renal disease.^6^

Down syndrome (DS), also known as trisomy 21, is a chromosomal condition caused by the presence of a full or partial extra copy of chromosome 21. It corresponds to a prevalence of approximately 8.27 individuals per 10,000 population and is recognized for its elevated incidence of specific conditions such as cardiac anomalies,^7^ hypothyroidism, type 1 diabetes mellitus,^8^ leukaemia,^9^ auditory impairments, and early-onset Alzheimer’s disease.^10,11^ However, the potential link between trisomy 21 and gout remains unexplored in existing literature. Previous case reports suggest an association may exist between trisomy 21 and gout, with a mean age of onset between 20 and 30 years.^12–15^ There are no large-scale studies demonstrating the relationship between trisomy 21 and gout. This study aims to establish an association between gout and DS using a large population-based aggregated electronic health record data (EHR) by comparing a cohort of individuals with trisomy 21 with individuals without.

## METHODS

### Data source and study design

An observation at our institution suggested a potential association between trisomy 21 and gout. To further investigate this relationship, we conducted a retrospective study using the TriNetX platform, which collects, de-identifies, and aggregates EHR data from healthcare organisations (HCOs) globally. The study utilised data on over 100 million patients in the U.S. Collaborative Network, representing over 60 HCOs. The TriNetX platform follows Health Insurance Portability and Accountability Act (HIPAA) and General Data Protection Regulation (GDPR) guidelines for de-identification. Studies using the TriNetX platform in this manner are considered non-human subjects research based on platform-wide regulatory determinations and expert attestation. Therefore, Institutional Review Board approval was not required. Strengthening the Reporting of Observational Studies in Epidemiology (STROBE) guidelines were followed.^16^ We examined the association between diagnoses of trisomy 21 and gout in this large dataset.

### Participants

**Figure 1.**
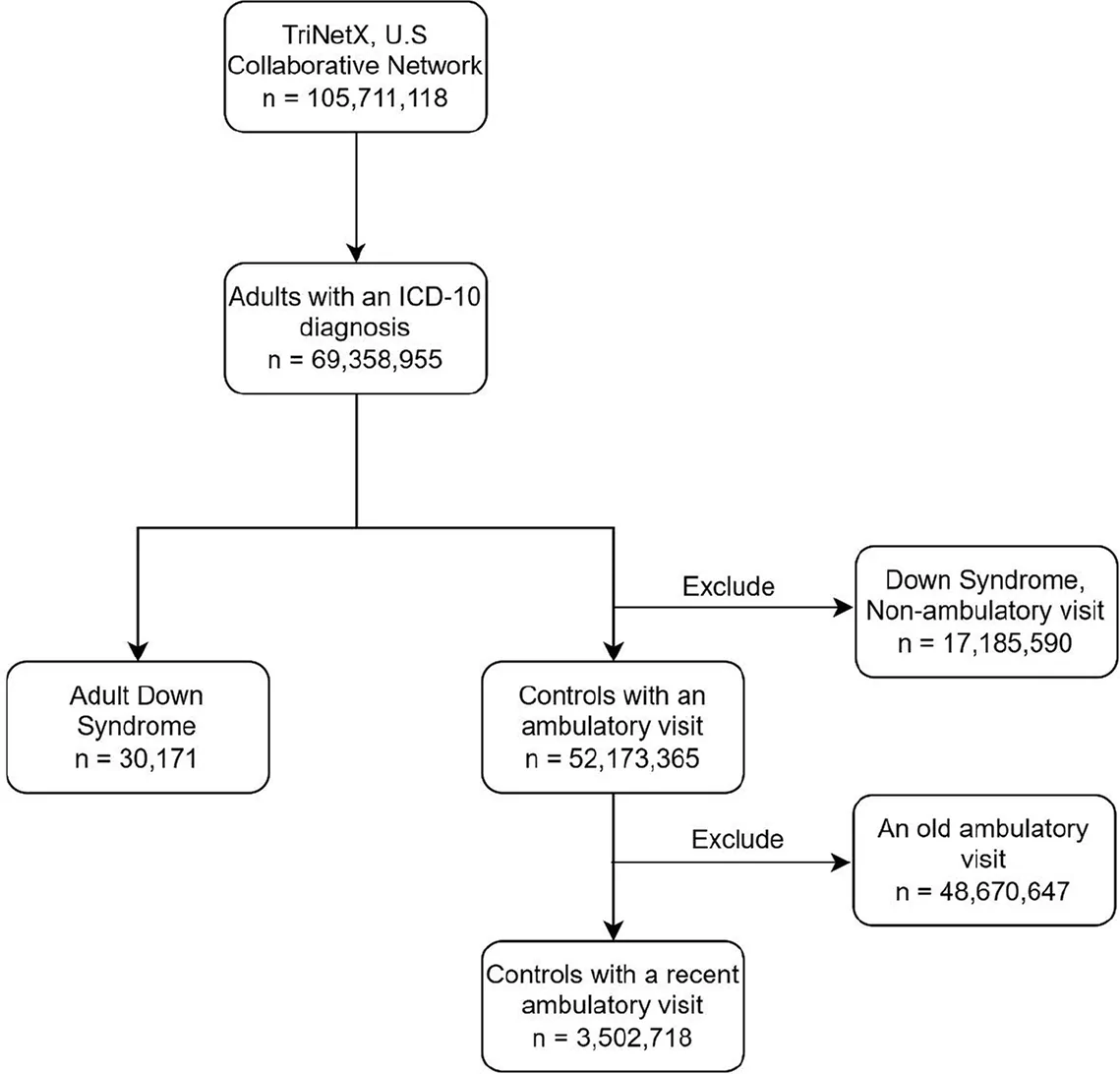
SCFA metabolism and immune modulation.

The study included two independent cohorts of adults aged 18 years or older: a trisomy 21 cohort and a non-trisomy 21 control cohort. Participants were identified based on International Classification of Diseases, 10th Revision, Clinical Modification (ICD-10-CM) diagnosis codes. The trisomy 21 cohort included individuals with at least one ICD-10-CM encounter diagnosis code for trisomy 21 (ICD-10-CM: Q90.0 [Trisomy 21, nonmosaicism (meiotic nondisjunction)], Q90.1 [Trisomy 21, mosaicism (mitotic nondisjunction)], Q90.2 [Trisomy 21, translocation], or Q90.9 [Down syndrome, unspecified]). Controls were excluded if they carried any of these codes. The non-trisomy 21 control cohort included individuals with at least one encounter diagnosis code for any condition other than trisomy 21 and at least one ambulatory visit within 5 months prior to the analysis date (December 22, 2023). Cohort selection is illustrated in **Figure 1**. A complete list of ICD-10-CM codes utilised is provided in **Supplementary Table S1**.

### Statistical methods

Patient with gout was defined as a patient having at least one encounter diagnosis of gout (ICD-10-CM M10.9) or chronic gout (ICD-10-CM M10.0) within the 20-year eligibility period prior to the index date (December 2022). Gout prevalence was stratified by age, sex, and race. The prevalence of gout was compared between the trisomy 21 and non-trisomy 21 control cohorts before and after propensity score matching for multiple factors in **Table 1**, including age, sex, race, comorbidities, and diuretic use. Within the cohort with trisomy 21, stratified analysis has been carried out to identify potential risk factors for gout; variables examined included age, sex, race, and body mass index (BMI). BMI was defined as the value recorded at the time of gout diagnosis. Chi-square test was used to compare gout prevalence between subgroups. The goal was to determine demographic and clinical factors associated with gout specifically in individuals with trisomy 21. The association between trisomy 21 and risk of gout was assessed by calculating RR with 95% confidence intervals (CI) according to Altman, 1991. Demographic data between cohorts was compared using two-sample t-tests for continuous variables and chi-square tests for categorical variables (significance defined as *p*<0.05). Race and ethnicity data were extracted from the electronic health records as recorded by participating healthcare organisations. Classification was based on patient self-report or administrative input using standardised categories aligned with U.S. federal definitions.

**Table 1.** Demographic and clinical characteristics of patients with and without trisomy 21 before propensity score matching.

**Characteristics**	**Before propensity matching**			**After propensity matching**						
	**Controls (N=3,502,718)**	**Trisomy 21 (N=30,171)**	**SMD**	**Controls (N=30,171)**	**Trisomy 21 (N=30,171)**	**SMD**
Age, years			**0.631**			0.003
18 – 30	1,040,765 (29.7%)	15,276 (50.6%)		15,273 (50.6%)	15,275 (50.6%)	
31 – 40	455,028 (13.0%)	5,362 (17.8%)		5,334 (17.7%)	5,362 (17.8%)	
41 – 50	406,814 (11.6%)	3,850 (12.8%)		3,861 (12.8%)	3,850 (12.8%)	
51 – 60	471,400 (13.5%)	4,063 (13.5%)		4,087 (13.5%)	4,063 (13.5%)	
61 – 70	452,210 (12.9%)	1,383 (4.6%)		1,384 (4.6%)	1,383 (4.6%)	
> 70	676,501 (19.3%)	237 (0.8%)		232 (0.8%)	237 (0.8%)	
Sex, %						
Female	1,977,320 (56.5%)	15,503 (51.4%)	**0.102**	15,453 (51.2%)	15,503 (51.4%)	0.004
Male	1,344,279 (38.4%)	14,114 (46.8%)	**0.171**	14,172 (47.0%)	14,113 (46.8%)	0.004
Unknown	181,119 (5.2%)	554 (1.8%)	**0.186**	546 (1.8%)	554 (1.8%)	0.000
Race, %						
White	2,304,329 (65.8%)	20,183 (66.9%)	**0.023**	20,164 (66.8%)	20,182 (66.9%)	0.002
Black/African	407,551 (11.6%)	2,984 (9.9%)	**0.055**	2,981 (9.9%)	2,984 (9.9%)	0.000
American						
Asian	133,856 (3.8%)	804 (2.7%)	**0.062**	797 (2.6%)	804 (2.7%)	0.006
Other	179,583 (5.1%)	1,722 (5.9%)	**0.035**	1,773 (5.8%)	1,722 (5.9%)	0.004
Unknown	477,399 (13.6%)	4,428 (14.7%)	**0.032**	4,456 (14.8%)	4,428 (14.7%)	0.003
Ethnicity, %						
Hispanic or Latino	320,482 (9.2%)	3,754 (12.4%)	**0.103**	3,770 (12.5%)	3,754 (12.4%)	0.003
Not Hispanic or Latino	2,484,148 (70.9%)	18,605 (61.7%)	**0.195**	18,629 (61.7%)	18,605 (61.7%)	0.000
Unknown	698,088 (19.9%)	7,812 (25.9%)	**0.142**	7,772 (25.8%)	7,811 (25.9%)	0.002
Comorbidities, %						
Hypertensive diseases	419,019 (12.0%)	2,323 (7.7%)	**0.143**	2,325 (7.7%)	2,323 (7.7%)	0.000
Diabetes mellitus	189,934 (5.4%)	2,102 (7.0%)	**0.067**	2,129 (7.1%)	2,102 (7.0%)	0.004
Chronic kidney disease	49,652 (1.4%)	858 (2.8%)	**0.099**	849 (2.8%)	857 (2.8%)	0.000
Nicotine dependence	94,832 (2.7%)	397 (1.3%)	**0.102**	402 (1.3%)	397 (1.3%)	0.000
Personal history of nicotine dependence	43,161 (1.2%)	288 (1.0%)	**0.019**	259 (0.9%)	288 (1.0%)	0.010
Transplanted organ and tissue status	7,020 (0.2%)	185 (0.6%)	**0.064**	173 (0.6%)	185 (0.6%)	0.000
Alcohol-related disorders	28,198 (0.8%)	88 (0.3%)	**0.069**	90 (0.3%)	88 (0.3%)	0.000
Medications, %						
Loop diuretics	56,395 (1.6%)	1,364 (4.5%)	**0.175**	1,332 (4.4%)	1,363 (4.5%)	0.005
Thiazide diuretics	124,439 (3.6%)	437 (1.4%)	**0.143**	421 (1.4%)	437 (1.4%)	0.000

Data are presented as count (percentage) or mean ± standard deviation (SD). SMD: standardised mean difference; BMI: body mass index.

## RESULTS

### Characteristics of the study population

The study encompassed a total of 3,532,889 individuals, including 30,171 participants diagnosed with trisomy 21 and 3,502,718 non-trisomy 21 control cohorts **(Figure 1)**. As detailed in **Table 1**, there were significant differences in several baseline characteristics between these groups. The mean age of individuals with trisomy 21 was 33.5 years (±14.9 years), notably younger than the 46.1 years (±20.9 years) observed in the control group. About 50% of the trisomy 21 group were aged between 18 and 30 years, compared to less than 30% in the control group. Only a marginal fraction of 0.8% of the trisomy cohort was over 70 years old, significantly lower than 19.3% among controls. Additionally, the average BMI for the trisomy group was 29.6 kg/m^2^ (±7.4), slightly higher than the 28.0 kg/m^2^ (±6.6) recorded for controls.

In terms of comorbidities, the trisomy 21 group exhibited lower prevalence rates of hypertensive diseases (7.7% vs 12.0%) and alcohol-related disorders (0.3% vs 0.8%) compared to controls. Conversely, higher incidence rates were noted for diabetes mellitus (7.0% vs 5.4%), chronic kidney disease (2.8% vs 1.4%), and transplanted organ status (0.6% vs 0.2%), all of which are key risk factors for developing gout. Moreover, the usage of loop diuretics was nearly threefold higher in the trisomy 21 group (4.5% vs 1.6% of controls), representing another potential contributing factor.

### Matched cohorts via propensity scores

Propensity score matching was implemented to align the baseline characteristics of trisomy 21 and control cohorts, aiming to reduce potential confounding in the analysis of gout outcomes. Post-matching, the age distribution was closely aligned across both cohorts, with approximately 50.6% of each group aged between 18 and 30 years. Gender distribution was also similar, with females representing 51.4% in the trisomy 21 group and 51.2% in the control group. Furthermore, rates of comorbid conditions and medication use associated with gout risk, including hypertension, diabetes, chronic kidney disease, and loop diuretic use, were comparably distributed (all *p*>0·05), **Table 1**.

The standardised mean differences (SMDs) demonstrated that propensity score matching effectively normalised the distribution of baseline covariates between the cohorts, with all parameters displaying SMDs below the 0.1 threshold, indicating adequate alignment between groups.

### Risk of gout diagnoses in individuals with trisomy 21 diagnoses

Prevalence of gout in the study population before propensity score matching. The frequency of individuals with a gout-related ICD-10-CM encounter diagnosis was compared between the trisomy 21 (n=30,171) and non-trisomy 21 (n=3,502,718) ICD-10-CM encounter diagnosis groups. Gout diagnoses occurred in 2.9% (n=875) of individuals with trisomy 21 diagnoses and 2.2% (n=79,086) of control subjects. In the matched cohorts after propensity score matching, gout diagnoses occurred in 2.9% (875 out of 30,171) of individuals with DS diagnoses and 1.1% (317 out of 30,171) of control subjects (p < 0.001), corresponding to a relative risk of 2.76 (95% CI 2.43–3.14. In unadjusted analysis, individuals with trisomy 21 had a 29% increased risk of developing gout disease compared to controls (RR=1.29, 95%CI=1.21–1.37, *p*<0.001).

### Heightened gout diagnoses in trisomy 21 diagnosed patients across demographics

Among individuals with trisomy 21 diagnoses, 2.90% (n=875) had ICD-10-CM gout encounter diagnoses, contrasted with 1.05% (n=317) in the matched general population cohort (*p*<0.001), as detailed in **Figure 3**. Stratified analysis by key demographic factors revealed consistently higher gout prevalence in the trisomy 21 cohort across all examined subgroups, including age, sex, and race. Analysing age categories, trisomy 21 individuals exhibited significantly elevated gout prevalence spanning young adulthood through over 70 years old. Specifically, 0.94% of those aged 18–30 years old with a trisomy 21 diagnosis had a gout diagnosis, compared to 0.26% of matched controls (*p*<0.001). Additionally, 5.78% of those aged 51–60 years with a trisomy 21 diagnosis had a gout diagnosis versus 2.10% of controls (*p*<0.001). This pattern persisted across genders, with males exhibiting a 5% gout diagnosis compared to 1.8% in controls and females 0.9% versus 0.4%, respectively (*p*<0.001). Racial disparities showed similar trends (**Table 2**). The only exception was individuals over 70 years old, where gout diagnoses did not significantly differ between patients with trisomy 21 diagnoses and controls (*p*=0.32).

**Figure 2.**
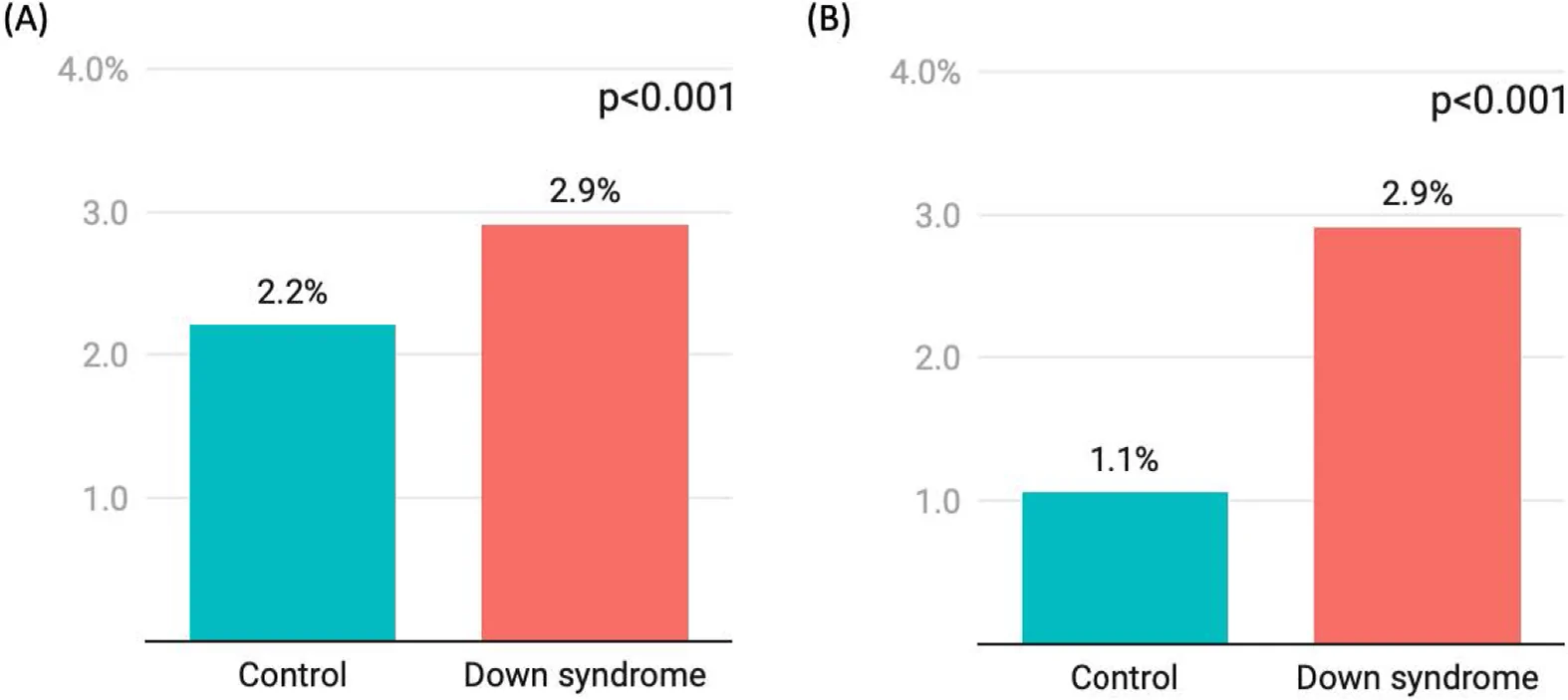
Prevalence of gout in study populations. **(A)** Prevalence of gout in the study population before propensity score matching. **(B)** Prevalence of gout in the matched cohorts after propensity score matching.

**Figure 3.**
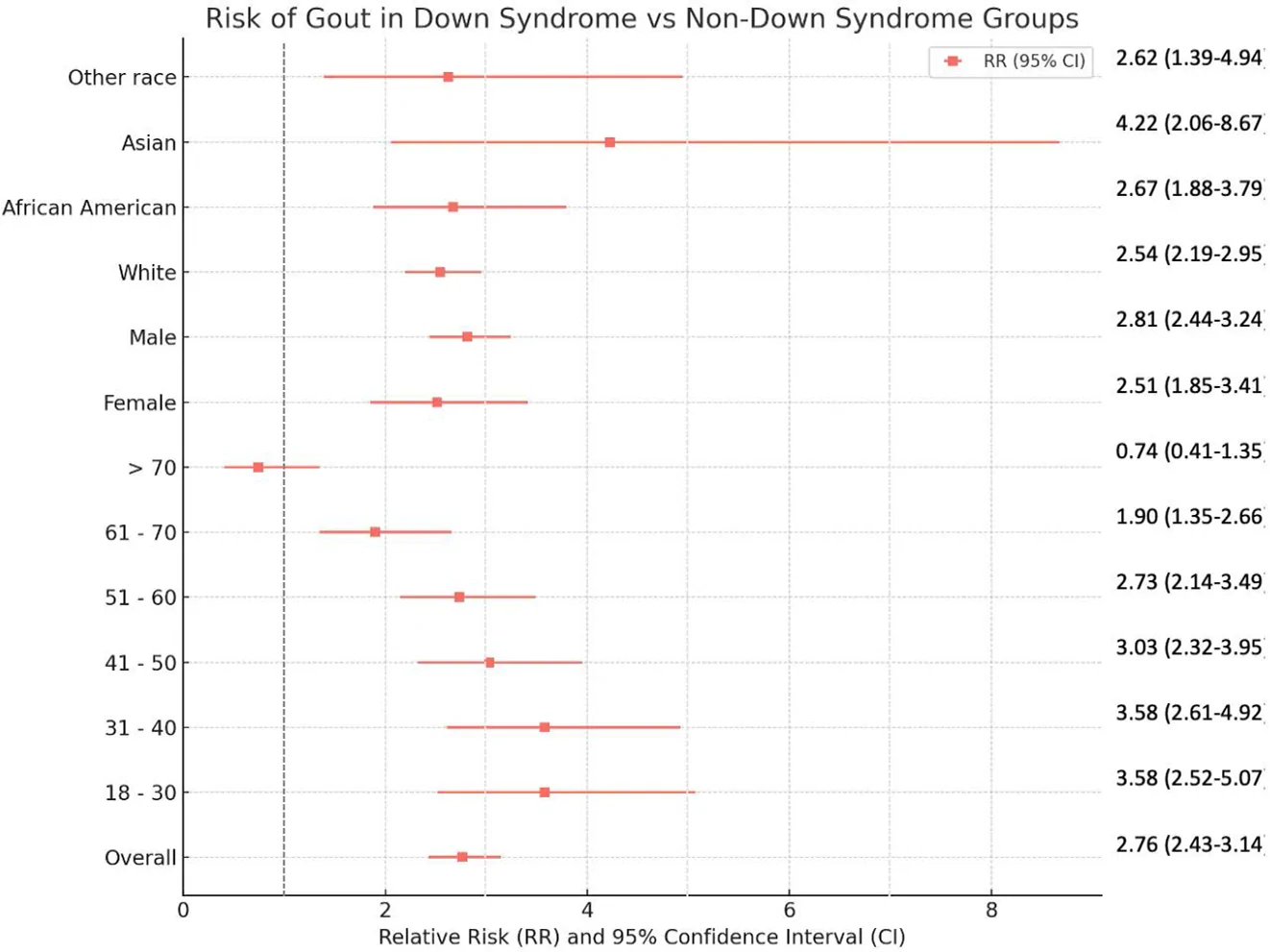
The relative risk (RR) and 95% confidence intervals (CI) are shown to demonstrate the risk of developing gout in individuals with trisomy 21 compared to controls across various demographic subgroups, including age, gender, and racial groups. Each bar represents the RR with a reference line at RR=1 (no increased risk).

**Table 2.** Comparative prevalence of gout between matched control and trisomy 21 cohorts.

**Characteristics**	**Controls**		**Trisomy 21**		**SMD**
	**Gout/Total**	**Percentage**	**Gout/Total**	**Percentage**	
Age in years					
18–30	(40/15273)	0.26	(143/15275)	0.94	0.091
31–40	(48/5334)	0.90	(172/5362)	3.21	0.170
41–50	(71/3861)	1.84	(215/3850)	5.58	0.205
51–60	(86/4087)	2.10	(235/4063)	5.78	0.195
61–70	(49/1384)	3.54	(93/1383)	6.72	0.146
> 70	(23/232)	9.91	(17/237)	7.17	−0.098
Sex					
Female	(252/15453)	1.63	(709/15503)	4.57	0.175
Male	(57/14172)	0.40	(143/14113)	1.01	0.075
Race					
White	(233/20164)	1.16	(592/20182)	2.93	0.129
Black	(42/2981)	1.41	(112/2984)	3.75	0.152
Asian	(9/797)	1.13	(38/804)	4.73	0.225
Ethnicity					
Hispanic or Latino	(25/3770)	0.66	(43/3754)	1.15	0.051
Not Hispanic or Latino	(228/18629)	1.22	(583/18605)	3.13	0.134

Data are presented as count (percentage). SMD: standardised mean difference.

### Elevated gout diagnoses in patients with trisomy 21 diagnoses across demographics

Trisomy 21 diagnoses were associated with consistently heightened gout diagnoses irrespective of demographic factors. Overall, the RR for having a gout ICD-10-CM encounter diagnosis in individuals with a Trisomy 21 ICD-10-CM encounter diagnosis was 2.76-fold (95% CI=2.43–3.14) higher than in controls, indicating a nearly threefold increased likelihood (**Figure 3**).

### Risk Factors for gout diagnoses within patients with trisomy 21 diagnoses

Within the cohort with trisomy 21 ICD-10-CM encounter diagnoses, higher age, male sex, Black or Asian race, and elevated BMI at the time of gout diagnosis were significant risk factors for gout ICD-10-CM encounter diagnoses (all *p*<0.05). Specifically, the risk of a gout diagnosis increased progressively with age, peaking in individuals over 70 years (RR 7.75, 95%CI 4.77–12.6), male sex (RR 5.45, 95%CI 4.56–6.51), Black race (RR 1.28, 95%CI 1.05–1.56), Asian race (RR 1.62, 95%CI 1.17–2.23), and morbid obesity - defined as a BMI of 40 or higher (RR 2.36, 95%CI 1.58–3.52) as shown in **Table 3.**

**Table 3.** Gout risk by demographics and clinical factors among patients with trisomy 21.

**Characteristics**	**Levels**	**RR**	**95% confidence interval**
Age, years	31–40 vs. 18–30 years	3.44	2.76–4.28
	41–50 vs. 18–30 years	6.00	4.87–7.39
	51–60 vs. 18–30 years	6.18	5.03–7.59
	61–70 vs. 18–30 years	7.18	5.56–9.26
	> 70 vs. 18–30 years	7.75	4.77–12.60
Sex	Male vs. Female	5.45	4.56–6.51
Race	Black vs. White	1.28	1.05–1.56
	Asian vs. White	1.62	1.17–2.23
BMI*, Kg/m^2^	Overweight vs. normal weight	1.95	1.24–2.50
	Obesity class I vs. normal weight	2.01	1.41–2.88
	Obesity class II vs. normal weight	2.31	1.57–3.39
	Obesity class III vs. normal weight	2.36	1.58–3.52

RR, relative risk; BMI, body mass index.

* BMI was available for 30% of trisomy 21 cohort. The body mass index (BMI) values used in this study are categorised as follows: Normal weight is defined as a BMI of 18·5 to 24·9 Kg/m^2^. Overweight is classified as having a BMI between 25 and 29.9 Kg/m^2^. Obesity is further classified into three classes; Class I obesity is defined as a BMI of 30 to 34·9 Kg/m^2^, Class II obesity ranges from 35 to 39·9 Kg/m^2^, and Class III obesity is a BMI of 40 Kg/m^2^ or greater.

## DISCUSSION

This large retrospective analysis of EHRs revealed a robust association between trisomy 21 and gout ICD-10-CM encounter diagnoses compared to the general population. Individuals with trisomy 21 diagnoses had a threefold higher risk of a gout diagnosis at a younger age than controls without trisomy 21. Furthermore, the prevalence of gout diagnoses in the trisomy 21 cohort exceeded that in the peak gout age range of 40–70 years among controls. These findings remained consistent across sexes and races, trisomy 21 as independently associated with increased gout risk and early development of the disease. Our results align with prior case reports describing gout onset in younger trisomy 21 populations compared to the typical middle-age onset in the public. Among individuals with trisomy 21, gout risk started rising earlier, reaching its peak between ages 40 and 70. This distinct early gout profile suggests preventive efforts should target younger trisomy 21 populations, under age 40.

The pathophysiology of gout involves chronic deposition of monosodium urate crystals in the presence of elevated serum urate concentration resulting from overproduction and/or underexcretion of uric acid.^17^ In the literature, various studies have described the prevalence of hyperuricemia in individuals with trisomy 21.^18–20^ However, the aetiology of hyperuricemia in this population remains uncertain. Several clinical studies have documented elevated serum uric acid levels in individuals with trisomy 21.^19,20^ Although the precise mechanisms remain incompletely defined, altered uric acid metabolism and renal handling have been proposed as potential contributors. Lower serum creatinine levels often observed in individuals with trisomy 21 more likely reflect reduced muscle mass rather than enhanced renal function, meaning serum creatinine may not reliably estimate glomerular filtration in this population. Further research is needed to clarify the pathophysiologic pathways linking trisomy 21 with hyperuricemia and gout. An increase in the activity of adenosine deaminase (ADA) enzyme level leading to excessive production of uric acid among trisomy 21 individuals was described by Puukka et al.^20^ However, normal activity of several other enzymes involved in the purine pathway, such as hypoxanthine phosphoribosyl transferase (HPRT), adenine phosphoribosyl transferase (APRT), and phosphoribosyl pyrophosphate (PRPP) synthetase enzyme in trisomy 21 individuals, was demonstrated by Watts et al. Low haptoglobin levels and low apolipoprotein A1 levels leading to renal dysfunction and defective lipid metabolism, respectively, were other mechanisms postulated to cause gout in the trisomy 21 population in a study by Chen et al.^22^ A cross-sectional study assessing renal involvement in the trisomy 21 population aged between 1 and 24 years showed no significant difference in characteristics such as age, BMI, or creatinine, nor any significant differences in individuals with elevated uric acid and normal-range uric acid, and no increased incidence of renal anomalies.^23^ Although 80–90% of people with gout tend to have hyperuricemia, only 18% of people with hyperuricemia develop gout.^24^ Serum urate is a proven predictor of incidental gout in the general population,^25^ but the presence of high serum urate levels alone does not establish an association with gout in trisomy 21 patients. Further research could help describe the pathophysiology involved in the increased prevalence of gout in the trisomy 21 population.

This study has some limitations. Use of EHR encounter diagnoses ICD codes may introduce false positives and negatives. However, the significance of a trisomy 21 diagnosis and the relative rarity of gout likely minimise this concern compared to more common conditions. Additionally, we could not confirm crystal-proven gout, and many times gout is a clinical diagnosis without a joint aspiration, so even though the provider thought that a patient had gout and put in a gout encounter diagnosis, some of these encounter diagnoses could be false positive encounter diagnoses. Nevertheless, misclassification rates would likely be similar between cohorts. Additionally, diagnostic bias cannot be excluded. Individuals with trisomy 21 may have more frequent healthcare encounters compared with the general population, potentially increasing the likelihood of gout diagnosis and coding. Conversely, communication challenges or atypical symptom presentation in some individuals with trisomy 21 could contribute to under-recognition or delayed diagnosis of inflammatory arthritis. Differences in clinical thresholds for assigning a gout diagnosis in this population may also introduce detection bias. Therefore, the observed association should be interpreted with consideration of these potential surveillance factors. An additional limitation relates to reliance on ICD-10-CM encounter diagnoses to define gout without validation through crystal analysis, imaging confirmation, or serum urate levels. Although gout is frequently diagnosed clinically in routine practice without synovial fluid analysis, the use of administrative coding may introduce outcome misclassification. While such misclassification would likely be non-differential between cohorts, potentially biasing results toward the null, we cannot exclude residual diagnostic uncertainty. Sensitivity analyses using stricter definitions, such as repeated gout diagnoses or medication-based confirmation, were limited by the constraints of aggregated EHR data within the TriNetX platform. Despite propensity score matching for major demographic and clinical factors, residual confounding cannot be excluded. Detailed renal function parameters (such as estimated glomerular filtration rate), dietary intake patterns, alcohol consumption beyond diagnostic coding, and medications affecting urate metabolism were not fully captured within the constraints of aggregated EHR data. Additionally, un-measured or incompletely recorded variables may have influenced the observed association. Therefore, our findings should be interpreted as demonstrating an association rather than a causal relationship between trisomy 21 and gout. Additionally, our analysis focused on the prevalence and relative risk of gout diagnoses and did not capture measures of disease severity, frequency of flares, presence of tophi, gout-related hospitalisations, or treatment intensity. The aggregated structure of the TriNetX platform limited detailed evaluation of longitudinal disease burden and clinical outcomes. Therefore, while our findings demonstrate a significant association between trisomy 21 and gout diagnosis, they do not inform the severity or clinical course of gout in this population. Clinicians should interpret these findings as evidence of increased susceptibility rather than increased disease severity, and further studies are warranted to characterise gout-related morbidity and treatment patterns in individuals with trisomy 21. In conclusion, this is the first large-scale study to demonstrate a significant association between trisomy 21 and gout. This real-world data shows heightened gout susceptibility in trisomy 21 across demographics, suggesting biological intersections with gout pathogenesis. These results suggest that increased clinical vigilance may be warranted for early gout symptoms in trisomy 21 patients to optimise treatment and preventive care. Further research on mechanisms linking trisomy 21 with increased risk of gout development across age, sex, and race is warranted. Findings support tailored screening and preventive strategies for this vulnerable population.

## DATA SHARING STATEMENT

Data may be obtained from a third party and is not publicly available. The data collected for this study, consisting of deidentified participant data, are processed on an aggregate level at the cohort level through the TriNetX platform environment. The data are housed within the secure environment of the TriNetX platform and due to the terms of use and data protection agreements, cannot be transferred or made accessible outside of the platform. Due to the nature of this research, individual participant data is not obtained. Consequently, there are no provisions for sharing individual research participant data, Institutional Review Board (IRB) approval, or informed consent documents. The study protocol and statistical analysis plan are proprietary but may be made available upon a justified request, subject to approval by our study team and in compliance with Tri-NetX policies. Access to the aggregated data is strictly limited to researchers affiliated with institutions that hold existing agreements with TriNetX. These restrictions are enforced to uphold the confidentiality and integrity of the data within the confines of the platform’s operational and legal frameworks.

## PATIENT AND PUBLIC IMPROVEMENT

N/A.

## SUPPLEMENTARY TABLE 1

https://mjrheum.org/assets/files/supplementary/MJR-2025-0423_Supplementary-Table-S1.pdf
